# Impact of early glymphatic disorders on the development of vascular dementia

**DOI:** 10.1186/s12880-026-02284-5

**Published:** 2026-03-16

**Authors:** Shiyan Xie, Yi He, Zhihong Zhao, Xiaolei Zhang, Yue Chen, Xinhui Zheng, Wentao Xuan, Jiechai Lin, Jitian Guan, Zhuozhi Dai, Renhua Wu

**Affiliations:** 1 Department of Medical Imaging, Second Affiliated Hospital, Shantou University Medical College, Shantou, 515041 China; 2 Department of Ultrasound, Shantou Central Hospital, Shantou, 515031 China; 3 Department of Radiology, Shantou Central Hospital, Shantou, 515031 China; 4 Shantou University Medical College Affiliated Cancer Hospital, Shantou, 515031 China

**Keywords:** Cognitive decline, Dynamic contrast-enhanced magnetic resonance imaging, Glymphatic system, Vascular cognitive impairment, Vascular dementia

## Abstract

Early treatment can effectively ameliorate cognitive decline and slow disease progression in vascular cognitive impairment (VCI). Glymphatic system dysfunction may significantly contribute to the onset and progression of diverse neurodegenerative diseases. To investigate its influence on VCI, we used dynamic contrast-enhanced magnetic resonance imaging to observe the exchange of cerebrospinal fluid (CSF) and interstitial fluid (ISF) in the brains of vascular dementia (VD) model and SHAM rats, and used the Morris water maze and hematoxylin–eosin staining to confirm the predictive value of the glymphatic system for VCI. The clearance rate of the CSF contrast agent, Gd-DOTA, from the brain interstitial space was slower in the VD than in the SHAM group. Behavioral tests showed a robust negative correlation between VCI and retention of Gd-DOTA contrast agents in the hippocampal region of VD rats. Pathology revealed a significant positive correlation between the number of apoptotic neurons in the hippocampal CA1 region and Gd-DOTA retention in the hippocampal region of VD rats. The clearance of brain ISF was hindered in the model rats, indicating that glymphatic system damage contributed to cognitive impairment in this group. Our findings suggest that glymphatic system function may be a predictor of VD development.

**Supplementary Information:**

The online version contains supplementary material available at 10.1186/s12880-026-02284-5.

## Introduction

Age-related cognitive impairment currently poses a significant public health challenge, and may emerge as a leading factor in East Asia with its aging population. Changes in the cerebrovascular system play a pivotal role in cognitive dysfunction resulting from various pathologies, such as age-related Alzheimer’s disease, significantly affecting cognitive function [1]. Vascular cognitive impairment (VCI), caused by vascular pathology, leads to cognitive decline, which can range from mild cognitive impairment to dementia. VCI represents 20–40% of all dementia cases. Although vascular pathologies are common in older individuals, vascular dementia (VD) caused solely by vascular lesions is rare, and most patients with VD exhibit other pathologies, particularly Alzheimer’s disease (AD). VCI is characterized by a gradual onset and a chronic course. Implementing proactive early treatment measures can effectively alleviate the symptoms of cognitive decline in patients with VCI, slow disease progression, and enhance a patient’s quality of life and functional abilities [2–5].

Cerebrovascular pathology may act cumulatively or synergistically with neurodegenerative pathology in causing dementia. Possible VCI mechanisms include chronic cerebral blood flow dysregulation, blood-brain barrier leak, inflammation, and cardiovascular dysfunction associated with aging. A high vascular risk factor prevalence in VCI in midlife can predict cognitive decline and dementia later in life. Hypertension, high cholesterol, diabetes, and midlife smoking are linked to a 20–40% higher dementia risk. Managing these risk factors may be highly effective for managing and preventing VCI, and may involve various lifestyle interventions [6, 7].

The glymphatic (perivascular) system is a recently discovered waste elimination system within the brain [8–14]. Anatomically, this flow occurs within the perivascular space (Virchow-Robin space), which is the channel enclosed between the basement membranes of the vascular smooth muscle cells (or pericytes) and the astrocytic end-feet. Its dysfunction is associated with the accumulation of insoluble amyloid plaques, aging, and onset of neurodegenerative disorders, such as AD, and other neurodegenerative conditions [10, 15]. The glymphatic system hypothesis suggests that cerebrospinal fluid (CSF) enters the brain parenchyma along periarterial spaces, mixes with interstitial fluid (ISF), and facilitates the clearance of harmful substances as the resulting fluid is driven out along periarterial channels. This fluid carries metabolic waste from the brain to the cerebrospinal fluid (CSF) reservoir and eventually drains through the meningeal lymphatics into the cervical lymphatics. Malfunction in this system is believed to impact the clearance of brain metabolites, such as amyloid-β (Aβ) and tau proteins [9, 16, 17]. The glymphatic system is affected by vascular pulsation, sleep [18–21], age [9, 22], and other factors.

A previous study discovered that injecting cholesterol crystals directly into mouse brains caused numerous small infarctions, damaging the brain’s glymphatic system [23], with the extent of glymphatic system damage and neural tissue inflammatory changes being more pronounced in aged than in young mice. This implies that cerebral microinfarctions worsen glymphatic system dysfunction, leading to a higher likelihood of protein and solute accumulation, which then remain confined within the microinfarction area, fostering protein aggregation and neural inflammation. Impaired glymphatic system functioning after stroke can decrease solute removal from the infarct area and nearby tissues, prolonging solute retention in the brain tissue, potentially culminating in neurodegenerative alterations and cognitive deficits, and increasing dementia risk [24]. In mouse ischemic stroke models, impaired glymphatic function and significant deposition of Aβ proteins have been observed in the infarcted hemisphere. Moreover, bilateral carotid artery stenosis in mice impaired glymphatic system function and increased the number of reactive astrocytes [7]. This damage also affected dermal artery pulsation in mice. Another study also showed a decrease in the Aβ and chondroitin clearance rate in APP/PS1 AD-model mice, suggesting that reduced ISF flow might enhance interactions between Aβ and its receptors, thereby influencing its clearance [25].

These studies suggested that damage to the glymphatic system in neurological diseases may worsen neurodegenerative changes and cognitive impairment. Evaluating and enhancing glymphatic system function could therefore potentially reduce neurological disease risk [26–28]. Two-photon laser scanning microscopy is an excellent tool for classifying glymphatic system images and facilitates high-resolution imaging in rodents [12]. By using fluorescent tracers and gene labeling, detailed anatomical information of the CSF flow pathways in the brain parenchyma can be obtained. Real-time fluorescent imaging of the whole cortex enables real-time observation of cortical activity [29], but requires surgical removal of the skull. In vitro imaging after tracer injection can be utilized to investigate the distribution and transportation of metabolites at the cellular and molecular levels, in conjunction with immunohistochemistry techniques. However, this method cannot be used to monitor real-time dynamic alterations in living animals [22, 30].

Application of magnetic resonance imaging (MRI) technology in glymphatic system research provides a potentially sensitive and non-invasive method for the diagnosis and treatment of relevant diseases, with significant clinical implications [31]. Chemical-exchange saturation-transfer (CEST), diffusion-weighted imaging, arterial spin labeling (ASL), three-dimensional T2 MRI, and other technologies are widely used in noninvasive glymphatic system imaging. He et al. achieved dynamic imaging of structural and metabolic changes in the living animal brain using CEST-MRI technology with an intravenous injection of Angiopep-2 [32]. Chen et al. utilized the lymphatic CEST method to evaluate glymphatic system function and successfully distinguished lymph, blood, and CSF in vivo [33]. Despite the advantages of minimal invasiveness and short observation time of CEST technology, further improvements in anatomical structure resolution and specificity are needed to minimize interference signals.

Other MRI techniques also contribute to this field. For instance, diffusion-weighted imaging (DWI) and arterial spin labeling (ASL) offer indirect insights into fluid motion and perfusion that may relate to glymphatic function, though they do not directly assess metabolite clearance [34, 35]. MRI has also been used to quantify the expansion of perivascular spaces and revealed an association with increased aortic stiffness. A recent study has used 3D T2-FLAIR MRI to reveal the structure of human dural lymphatics and discovered age-related changes. Although these methods allow visualization of the glymphatic system structure, functional monitoring remains limited [36].

In recent years, T1-weighted MRI has been applied to assess glymphatic function in patients with nervous system diseases [37, 38]. Iliff et al. achieved long-term observation of brain tissues and deep structures in a preclinical model by injecting gadolinium contrast agents into the cisterna magna combined with dynamic contrast-enhanced MRI techniques [39]. Importantly, these findings from animal models have been translated to humans. A pivotal study by Ringstad et al. used intrathecal gadobutrol and MRI to provide in vivo evidence in humans that a CSF tracer has access to all brain subregions, and its clearance was delayed in a cohort of patients with dementia, directly linking impaired glymphatic clearance to human neurodegenerative conditions [40]. Alternatively, a less invasive method is imaging of brain meningeal lymphatic vessels through T1 mapping using intravenously administered contrast agents [41].

In parallel, advanced MRI techniques continue to emerge, offering diverse options for research on the glymphatic system. For example, dynamic glucose-enhanced MRI has been used to evaluate impairment of the glymphatic system in AD model rats, demonstrating the potential of this method for early AD stratification [42]. Overall, these and other emerging MRI techniques hold tremendous potential in neuroscience [43, 44].

The pathological mechanisms underlying VCI are not fully understood. Furthermore, the specific role and functional status of the glymphatic system in the onset and progression of VCI remain to be fully elucidated.

Therefore, we used 7-T MRI to evaluate glymphatic system function in a rat model, performed behavioral experiments, and correlated the results with those of in vitro studies, to determine whether glymphatic imaging could be used to predict VCI, to guide timely implementation of interventions and preventive measures for high-risk patients.

## Materials and methods

The study utilized rats obtained from the Animal Center of Shantou University School of Medicine in Guangdong, China. Anesthesia was induced in rats using 4.0% isoflurane, followed by a maintenance dose of 1.5–2.5% isoflurane. Throughout the experiment, rat body temperature and respiration were continuously monitored using an MRI-compatible small-animal monitoring system (SAI Technologies, Memphis, TN, USA).

Supplementary Figure 1. Schematic diagram of the experimental sequence. The flowchart illustrates the major procedures in the study, including animal model establishment, magnetic resonance imaging (MRI), behavioral testing in the Morris water maze, and pathological analysis.

### Animal model and groups

Male Sprague–Dawley rats weighing 250–300 g and aged 2–3 months were randomly assigned to either the VD model group or the sham-surgery (control, SHAM) group. The rats were maintained in an environment with a natural magnetic field. The humidity was kept at 50 ± 10%, with a temperature of 22 ± 1 °C.

The VD model group was established by ligating the bilateral carotid arteries. The rats were anesthetized with 4% isoflurane, which was subsequently reduced to 2.5–3%. The rats were positioned supine on the surgical table during the procedure, with their abdomens facing upward. The surgical area was shaved and cleaned with distilled water and iodine solution. Next, a midline incision was made in the neck and the skin and subcutaneous tissues were opened longitudinally. The neck muscles were bluntly dissected, and the bilateral carotid artery sheaths were exposed, taking care to prevent injury to the vagus nerve. In the VD group, the carotid arteries were doubly ligated with 3–0 silk sutures and were then divided in the middle to obstruct the arterial blood flow. The neck muscles were returned to their normal anatomy, and the skin was sutured with 0 silk sutures and disinfected. The SHAM group underwent similar treatment, except that the bilateral carotid arteries were separated and sutured without ligation or division.

After surgery, the animals were randomly assigned to three independent experimental cohorts to undergo different endpoint assessments. The MRI Cohort received an intricisterna magna (ICM) injection of Gd-DOPA and was subsequently subjected to MRI. The Behavior and Histology Cohort underwent behavioral tests, including the Morris water maze, followed by perfusion and sacrifice for brain collection. The brain tissues were then processed for conventional histopathological staining including H&E, Nissl, and LFB. The Immunofluorescence Cohort received an ICM injection of fluorescent tracers, followed by perfusion and sacrifice. The brains were collected for immunofluorescence staining and analysis.

### MRI experiments

All experiments were performed using a 7.0-T horizontal bore (16-cm) small-animal MRI scanner (Agilent Technologies, Santa Clara, CA, USA). Standard 9563 coils from Agilent Technologies (Santa Clara, CA, USA) and surface coils from Time Medical Technologies (Shanghai, China) were used for transmission and reception during imaging.

CSF–ISF exchange was assessed using 3D T1-weighted MR images (T1WIs) and a Gd-DOTA contrast agent. Each MRI session employed a 3D gradient-echo sequence for animal imaging. The ge3D protocol used a repetition time of 15 ms, echo time of 4 ms, flip angle of 15°, average field-of view (FOV) of 35 × 35 × 35 mm^3^ and a 128 × 128 × 128 matrix. Dynamic 3D T1WIs were acquired continuously for 5 h. The acquisition protocol included two baseline scans, followed by the administration of intra-cisterna magna (ICM) Gd-DOTA contrast agent (21 mMDOTA) via an indwelling catheter, during MRI. Eighty microliters of the paramagnetic contrast agent was delivered intrathecally at an infusion rate of 1.6 μL per min, with a total infusion time of 50 min.

### Morris water maze

The behavioral performance of the rats was evaluated using the Morris water maze [45]. The experiment utilized a water pool divided into four quadrants, which was equipped with a tracking and analysis system and real-time recording devices. The test consisted of three consecutive stages: the visible platform test, hidden platform test, and probe trial. In the visible platform test, rats were required to find an above-water visible platform. The hidden platform test assessed rats’ spatial learning ability as they located the submerged platform without clear cues. In the probe trial, memory was measured by tracking the time and distance spent in the quadrant where the platform was placed. The rats were acclimated to the setting before the start of the experiment. The pool was filled with water containing non-toxic black dye for tracking, partitioned into quadrants with reference markers, and tests were conducted over 6 days with intervals of over 20 min between each test, with each stage having specific procedures and time limits.

### Hematoxylin–eosin staining

After completing all the experiments, the rats were anesthetized and perfused with 50 mL of saline and 50 mL of 4% paraformaldehyde through the left ventricle of the heart. Following this euthanasia, rats were decapitated, brain tissue was extracted, stored in 4% paraformaldehyde for 24 h, dehydrated, embedded in paraffin, sectioned, and histologically examined. Specifically, brain tissue was sectioned axially into 5-μm-thick slices, then stained with hematoxylin and eosin (HE) at 50 °C for 45 min. All sections were examined under a microscope.

### Nissl staining

Nissl staining was used to assess neuronal damage in the nervous system. Firstly, tissues are sliced into 25-μm-thick sections, transferred to phosphate-buffered saline (PBS) buffer for flattening, and then air-dried onto glass slides. Subsequently, they were baked at 60 °C in an oven for 60 min, dehydrated, and immersed in a solution of Cresyl violet for 1 h. After staining, the sections were gently rinsed and dehydrated until the background stabilized; they were then dehydrated, cleared, and mounted for examination under a microscope to evaluate neuronal features, including Nissl bodies, and to assess nervous system damage.

### Luxol fast blue staining

Luxol Fast Blue (LFB) staining was used for specific examination of myelin sheath morphology and structure. Sections were deparaffinized, soaked in xylene and a decreasing gradient of ethanol, and rinsing with distilled water. Next, the sections were immersed in LFB staining solution, washed, and differentiated using ethanol and lithium carbonate immersion to manage the staining duration until the gray matter lightened in color. Differentiation was repeated as necessary, followed by counterstaining with eosin, rinsing, air-drying, and mounting using a neutral medium.

### Brain tissue immunofluorescence staining

For immunofluorescence staining, 100-μm-thick coronal sections were cut, starting from the lateral ventricle, using a cryostat. The brain slices were then stored in 1× PBS solution and kept in a 4 °C refrigerator. For immunofluorescence staining, two brain slices were placed into each well of a 24-well plate, washed with 1× PBS, incubated in a permeabilization solution for 1 h, and then treated with a blocking solution for 1 h at room temperature. After washing, primary antibodies were added and sections incubated overnight at 4 °C, followed by washing with PBS, and incubation with secondary antibodies for 1 h. Finally, DAPI staining was performed and the slices were mounted with glycerol for imaging using confocal microscopy.

### Ex-vivo fluorescence imaging

On the 7th day after modeling, the drainage function of the meningeal lymphatic vessels in the VD and Sham groups was assessed. The animals were anesthetized with 4% isoflurane and placed in a prone position on the surgical table. The teeth of the rats and both external ear canals were secured to the stereotactic apparatus. The dorsal neck area was prepared by shaving and disinfecting with an iodine solution. A midline incision was made and the muscle layer was gently separated to expose the atlanto-occipital membrane. A 5-μL microinjector was inserted horizontally into the subarachnoid space at a depth of approximately 0.2 mm, and 3 μL of ethidium bromide (EB) solution was injected into the medullary cistern at a rate of 2.5 μL/min. After completion of the injection, the needle was left in place for 2 min before slowly withdrawing it to prevent CSF leakage. Layered suturing, disinfection, and fixation were then performed. The rats were euthanized by perfusion 60 min after injection. The brains were cryogenically stored at −70 °C. Brain sections of 4-μm thickness were obtained using a cryostat. DAPI was applied to the tissue sections, which were then stained for 3–5 min, rinsed with PBS, and sealed with neutral resin. The fluorescent sections were observed under a Pannoramic MIDI microscope.

### Data analysis

Based on the methodology of Iliff et al. [12], MRI scan-to-scan mismatches induced by head movements were corrected by manually aligning the images from each scan with the time-averaged (mean) image using the rigid registration tool in the RadiAnt DICOM Viewer software. All time-series images were subtracted from the baseline-averaged image and then divided by the baseline-averaged image to ensure that the voxel intensities represented the percentage change relative to the baseline-averaged image, using the following equation:

1 $$\mathrm{P}\left( \mathrm{i,j,k} \right){\rm{ }} = {\rm{ }}\left[ {\mathrm{I}\left(\text {i,j,k} \right){\rm{ }}-{\rm{ }}\mathrm{I}\_\mathrm{base}\left( \mathrm{i,j,k} \right)} \right]{\rm{ }}/{\rm{ }}\mathrm{I}\_\mathrm{base}\left( \mathrm{i,j,k} \right){\rm{ }} \times {\rm{ }}100$$

where P is the percentage change in the signal from baseline, i is the image intensity, and (i,j,k) is the voxel position. After 3D reconstruction of each data point using the aforementioned software (RadiAnt DICOM Viewer) running on Windows 10, and after manually adjusting to the same and appropriate window width and window position, the most centered sagittal section was selected. ImageJ software was used to quantify the image signal intensity. To facilitate visual comparison, the images were masked using ImageJ to isolate the brain tissue. This was performed manually on a slice-by-slice basis by a researcher blinded to the experimental groups, who delineated the brain region using the polygon selection tool, thereby excluding non-brain structures.

### Statistical analyses

Statistical analysis was performed using IBM SPSS Statistics version 19.0 (IBM SPSS Inc., Armonk, NY, USA) and GraphPad Prism 8.0.0 software (GraphPad Software Inc., San Diego, CA, USA). The primary variable for comparison was the percentage change of signal intensity from the baseline over time. Student’s t-test was used to compare two groups of data, and one-way analysis of variance was used for comparison of more than two groups. *p* < 0.05 was considered statistically significant. Data are presented as mean ± standard deviation.

## Result

### In vivo imaging

**Fig. 1:**
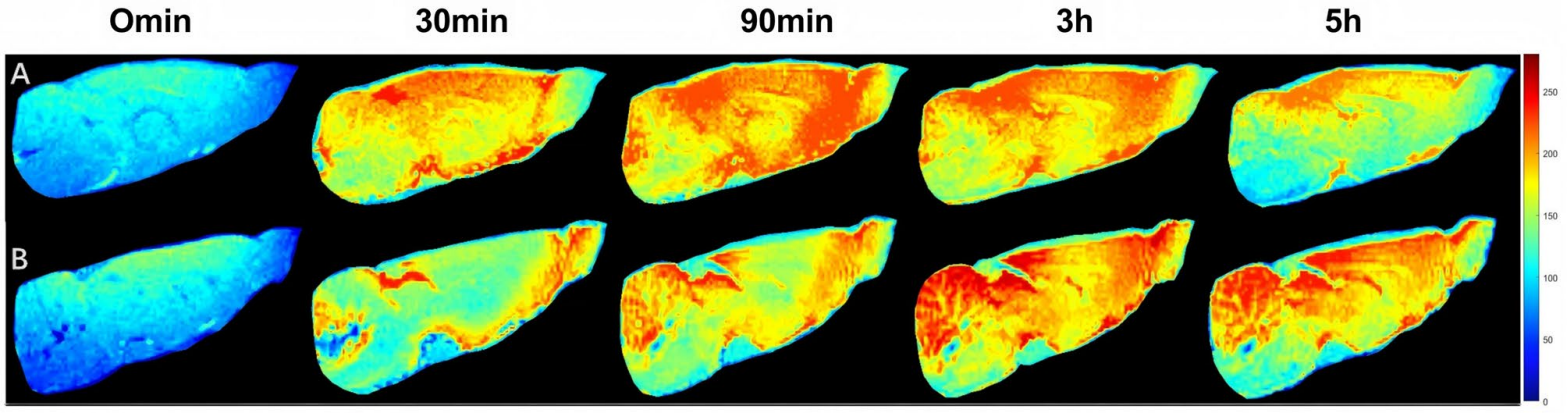
Dynamic contrast-enhanced magnetic resonance imaging of Gd-DOTA distribution in SHAM and VD rats. Representative in vivo T1-weighted images show the early influx (0 min, 30 min) and parenchymal enhancement at 90 min, 3 h, and 6 h after intra-cisterna magna injection of Gd-DOTA in (**A**) SHAM and (**B**) VD rats. The signal intensity, represented by the color bar, reflects the relative concentration of the contrast agent. Images were masked to display only the brain tissue region of interest

In vivo brain dynamic contrast-enhanced MRI a lower signal intensity DOTAin VD rats (Fig. 1B) at 30 and 90 min than that in SHAM rats (Fig. 1A). The signal intensity gradually increased after the ICM Gd-DOTA injection, peaking at approximately 3 h and slightly decreasing by 5 h.

However, both the 3-h and 5-h signals were higher in the VD than in the SHAM group. In contrast, the SHAM group displayed a gradual increase in signal intensity after injection, peaking at 90 min, followed by a gradual decrease.

Supplementary Figure 2. Location of the regions of interest (ROIs) used for analysis. Representative brain sections showing the anatomical locations of the ROIs analyzed in this study, including the hippocampus, cerebral cortex, and olfactory bulb (OB).

**Fig. 2:**
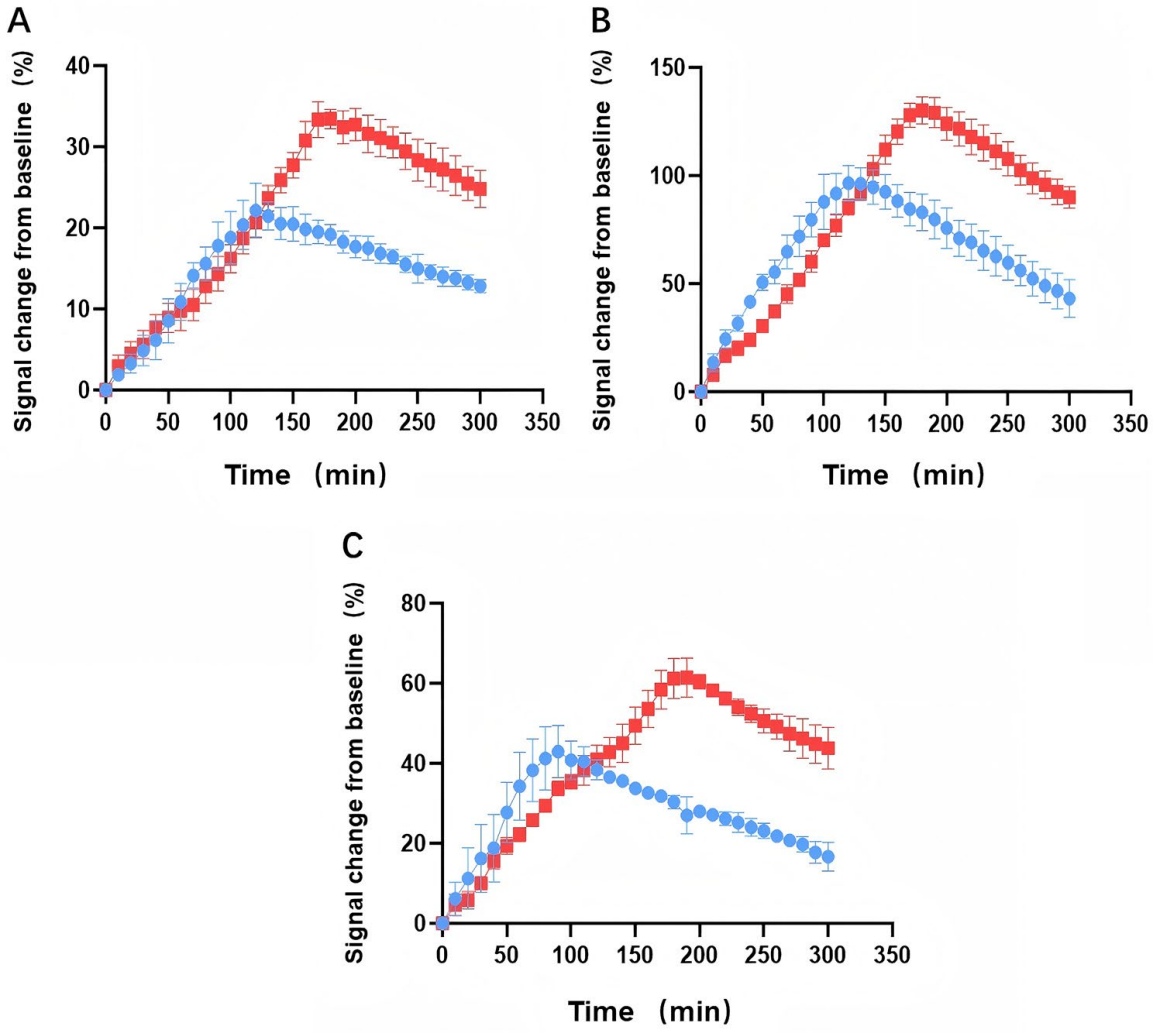
Quantitative time-course of signal evolution differences on magnetic resonance imaging in the brains of SHAM (blue) and VD (red) group rats (red): hippocampus (**A**), cortex (**B**), and olfactory bulb (**C**), *N* = 6 per group

Figure 2 shows the signal intensity evolution after contrast agent injection. In the hippocampal region (Fig. 2A), VD rats exhibited a gradual increase in signal intensity after contrast agent injection. The signal peaked at 180 min with a maximum value of 33.46%. In contrast, the SHAM group peaked at 120 min, with a maximum value of 22.14% (*p* = 0.035). In the cortical region (Fig. 2B), VD rats showed a progressive increase in signal intensity after contrast agent injection. The signal peaked at 180 min, at 130.13%, in contrast to the peak of the SHAM group at 120 min, which reached 96.59% (*p* = 0.046). In the olfactory bulb region (Fig. 2C), VD rats displayed a progressive increase in signal intensity after contrast agent injection. The signal peaked at 190 min at 61.4%, whereas that of the SHAM group peaked at 90 min at 42.96% (*p* = 0.002). These findings indicated reduced glymphatic system influx in the hippocampus, cortex, and olfactory bulb regions of the VD group as compared to the SHAM group.

At 5 h (300 min), signal intensity changes were observed. In the hippocampal region, the VD group showed a 24.82% change, as compared to a 12.86% change in the SHAM group. Similarly, in the cortical region, the VD group displayed a 90.04% change compared to 43.19% in the SHAM group. In the olfactory bulb region, the VD group exhibited a 43.8% change, whereas that in the SHAM group was 16.72%. These results suggest reduced glymphatic system efflux and contrast agent clearance in the VD group across various brain regions, leading to increased levels of residual contrast agent and subsequent signal intensity changes.

### Behavioral tests

**Fig. 3:**
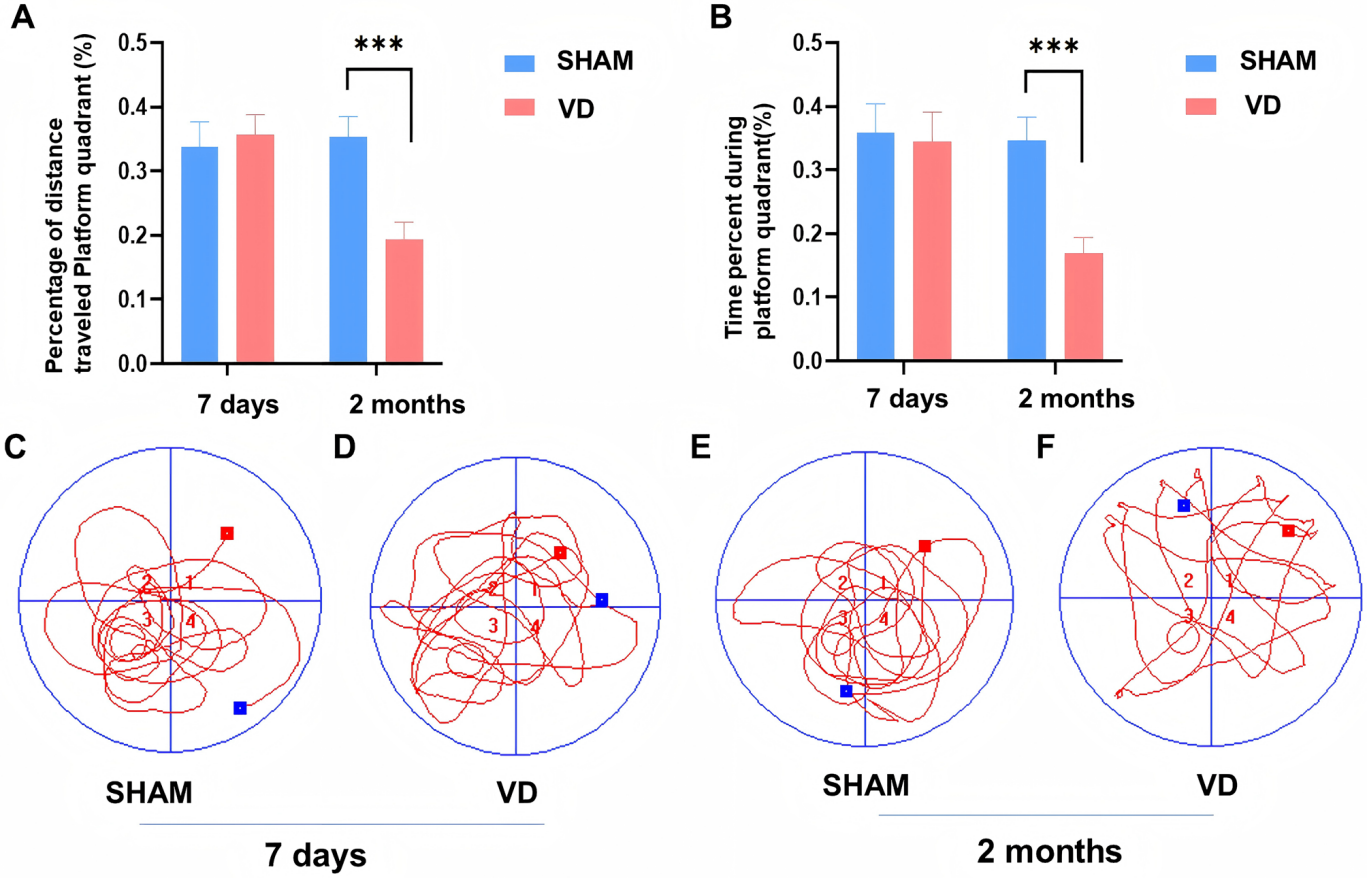
Results of the Morris water-maze test, *N* = 6 per group. (**A**) Ratio of distance traveled in the target quadrant for the 7-day post-modeling group and the 2-month post-modeling group (^***^p ＜ 0.001); (B) Ratio of time spent in the target quadrant for the 7-day post-modeling group and the 2-month post-modeling group (^***^p ＜ 0.001). (**C, D**) Plots of spatial exploration trajectories of the SHAM group versus the VD group at 7 days after modeling. (**E, F**) Plots of spatial exploration trajectories of the SHAM group versus the VD group at 2 months after modeling. A significant difference was observed between VD and SHAM rats only at 2-months post-modeling, in terms of both the target quadrant distance ratio and time ratio. This indicates that cognitive decline in the VD model rats manifested approximately 2 months after modeling (* *p* < 0.05, **p < 0.01, ***p < 0.001)

At 7-days and 2-months post-modeling, we used the Morris water-maze experiment to assess the hippocampal cognitive function of the rats in the two groups. At the end of the experiment, we compared the target quadrant path (Fig. 3A) and time (Fig. 3B) ratios between the two groups during the tracking phase. The trajectory diagram of the water maze experiment showed that the VD and SHAM rats in the 7-day group successfully navigated to the target quadrant and platform, indicating intact spatial memory. These images showed that rats at 7-days post-modelling did not yet exhibit spatial learning ability impairments, equivalent to the preclinical stage of cognitive decline in humans with VD. In contrast, the 2-month group displayed distinct patterns. VD rats exhibited random exploration behavior, whereas SHAM rats consistently navigated to the target quadrant and platform. During the tracking phase, the rats in the 2-months post-modeling group had a shorter path ratio in the target quadrant than did the SHAM rats (Fig. 3A, *p* < 0.001), and the time spent in target quadrant activity was also shorter than that of the SHAM rats (Fig. 3B, *p* < 0.001). This difference confirmed that VD rats in the 2-month group displayed erratic exploration behavior, and exhibited memory impairments or decline during the experimental process, suggesting that memory deficits in this VD rat model appeared approximately 2 months after modeling.

Pearson correlation analysis was used to analyze the ratio of the target quadrant dwell time ratio to the change in baseline signal intensity 5 h after contrast agent injection in the MRI experiment. The results showed a strong linear correlation between signal intensity and the target quadrant dwell time ratio (*r* = −0.7961, *p* = 0.002). Thus, the occurrence of cognitive impairment in the 2-months post-modeling VD rats in the water-maze experiments was consistent with the MRI-determined features of an impaired glymphatic system.

### Ex vivo analysis

**Fig. 4:**
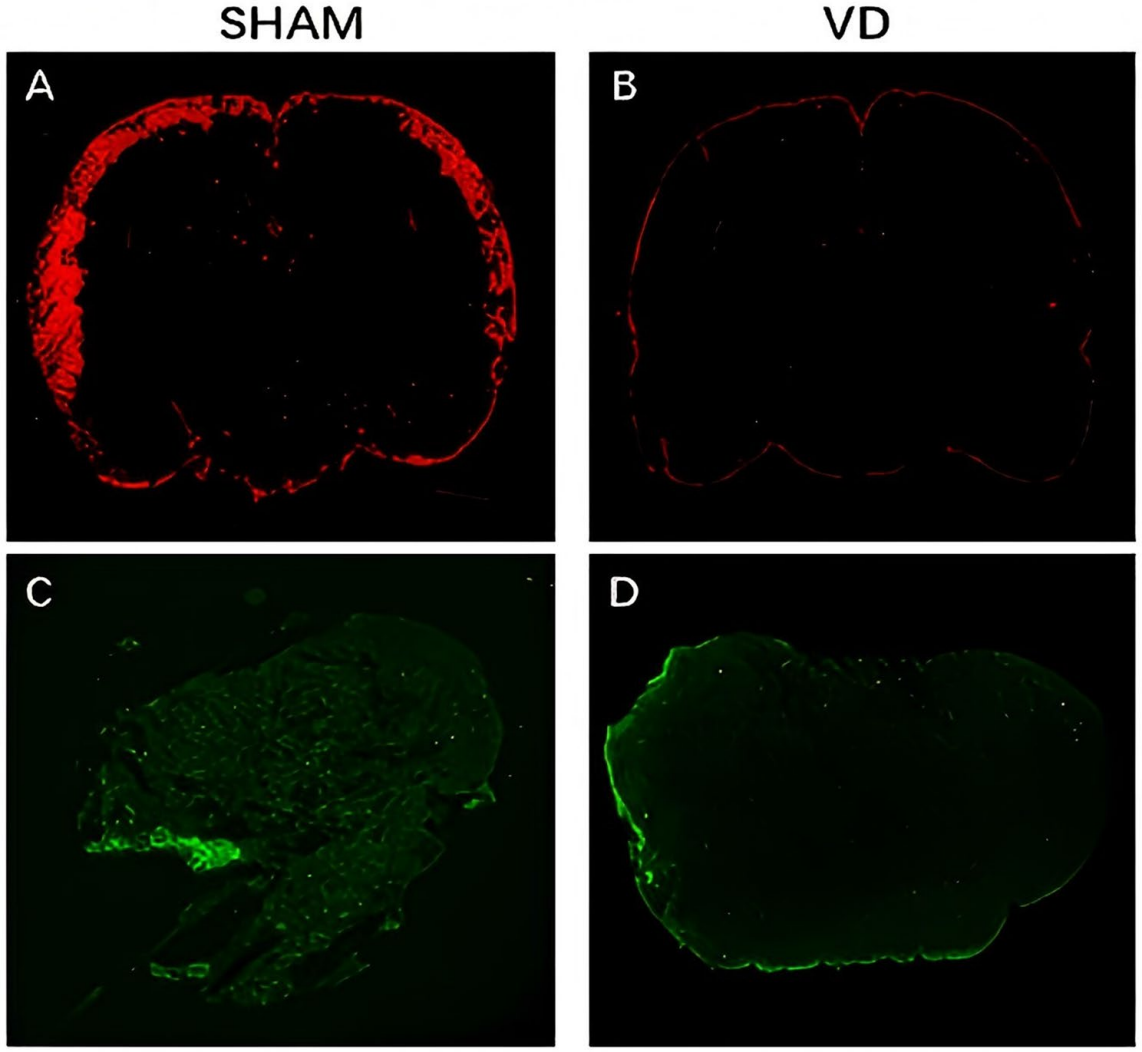
Ex vivo analysis (**A**) Fluorescence analysis of brain tissue in the SHAM group and (**B**) VD group. Fluorescence analysis of lymph node section in the (**C**) SHAM group and (**D**) VD group

Using ICM injection of EB fluorescent solution, we examined the function of the glymphatic system in the VD and SHAM groups at 7 days after modeling (Fig. 4). Compared with the SHAM group (Fig. 4A), rats in the VD group showed delayed meningeal lymphatic drainage at this time-point. The fluorescence-positive area of brain sections in the VD rats was reduced (Fig. 4B), suggesting that the inflow function of the glymphatic system in the VD rats was impaired. Moreover, relative to the fluorescence staining of lymph nodes in sections from SHAM rats (Fig. 4C), the fluorescence-positive area of the lymph nodes was reduced in the VD rats (Fig. 4D), suggesting that the outflow function of the glymphatic system in the VD rats was also impaired.

### HE staining

**Fig. 5:**
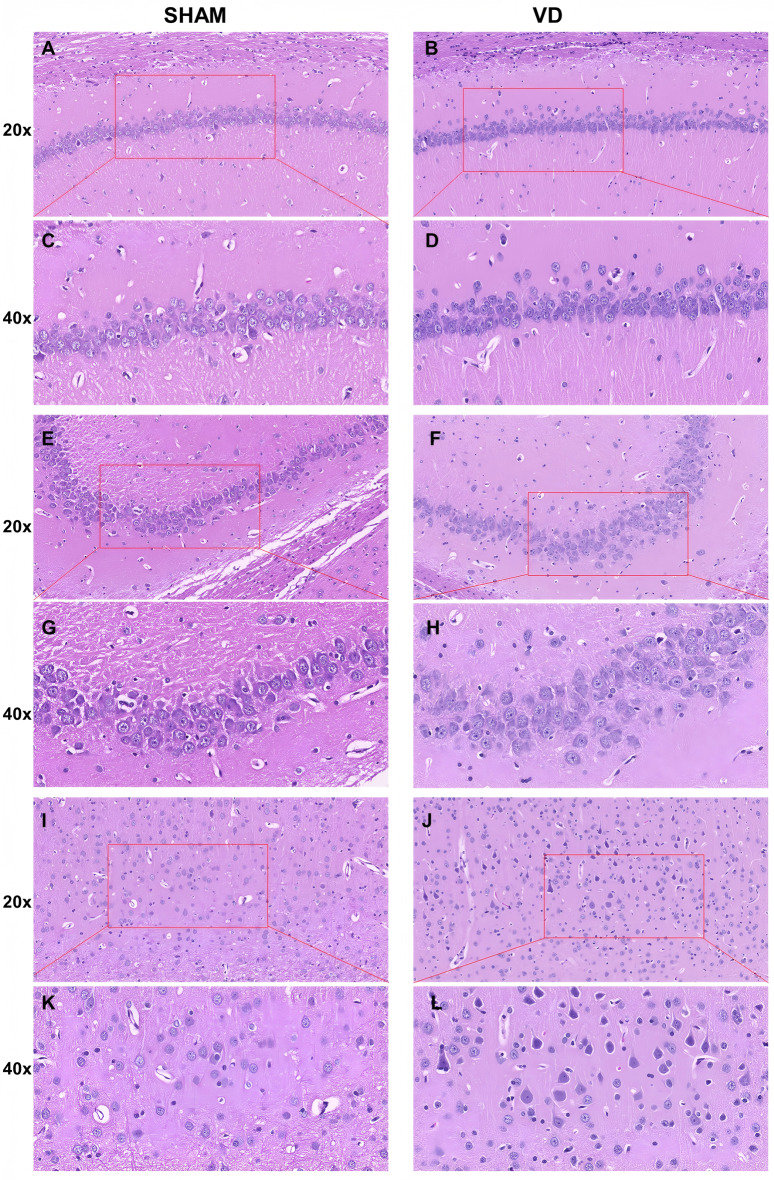
Hematoxylin–eosin (HE) staining of hippocampal CA1 region, CA3 region, and cortex. (**A, C**) HE staining of the CA1 area of the cerebral hippocampus in the SHAM group; (**B, D**) HE staining of the CA1 area of the cerebral hippocampus in the VD group; (**E, G**) HE staining of the CA3 area of the cerebral hippocampus in the SHAM group; (**F, H**) HE staining of CA3 area of the cerebral hippocampus in the VD group; (**I, K**) HE staining of cerebral hippocampus cortical area in the SHAM group; and (**J, L**) HE staining of cerebral hippocampus cortical area in the VD group; (**A, B, E, F, I,** and **J**: 20× magnification; **C, D, G, H, K**, and L 40× magnification)

The hippocampus is highly vulnerable to ischemia and hypoxia, and plays a crucial role in the central nervous system. The hippocampal CA1 region is primarily associated with memory formation and spatial cognition. VCI involves decreased cerebral blood flow, impacting hippocampal synaptic function and structure, and leading to neuronal apoptosis and subsequent learning and memory impairments. Hence, after the water-maze trials, the hippocampal structure, neuronal status, and perivascular spaces were evaluated by pathology. HE staining was performed on brain sections at the hippocampal level (Fig. 5). After 2 months, the VD group exhibited loosely arranged neuronal cells and increased cellular gaps in the CA1 and CA3 regions of the hippocampus. Neuronal apoptosis, characterized by varying levels of intense nuclear staining and cytoplasmic condensation, was observed in the CA1 and CA3 regions of the hippocampus and cortex (Fig. 5B,D). Conversely, neurons in the CA1 and CA3 regions of the SHAM group exhibited an orderly arrangement, consistent cell gaps, and intact cellular structures in the hippocampus and cortex (Fig. 5A,C). Statistical analysis (Fig. 7A) revealed a significant increase in neuronal apoptosis in the hippocampal CA1 region of VD rats post-2-month modeling, in contrast to SHAM rats.

**Fig. 6:**
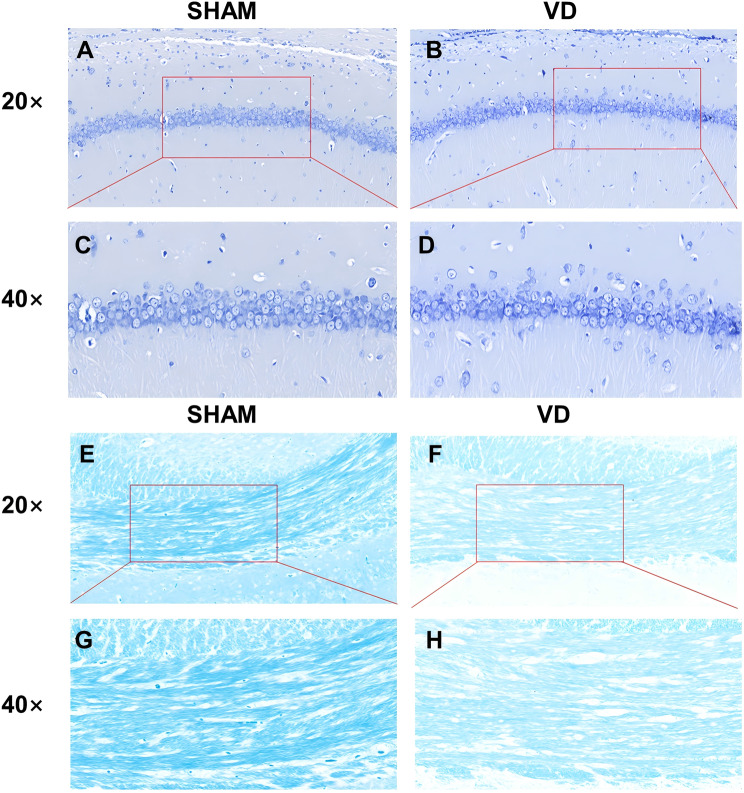
Nissl staining and Luxol Fast blue (LFB) staining of brain sections. Nissl staining of hippocampal CA1 region at 20× magnification in the in brain sections of (**A, B**) The SHAM group and VD group. (**C**) Magnification (40×) of the image shown in (**A**) for the SHAM group and (**D**) That shown in (**B**) for the VD group. (**E**) Regional map of the corpus callosum in LFB-stained sections of SHAM rat brain under 20× magnification. (**F**) Regional map of the corpus callosum in LFB-stained sections of VD rat brain under 20× magnification. (**G**) Magnification (40×) of the corpus callosum in LFB-stained sections of SHAM rat brain. (**H**) Magnification (40×) of the corpus callosum in LFB-stained sections of VD rat brain

**Fig. 7:**
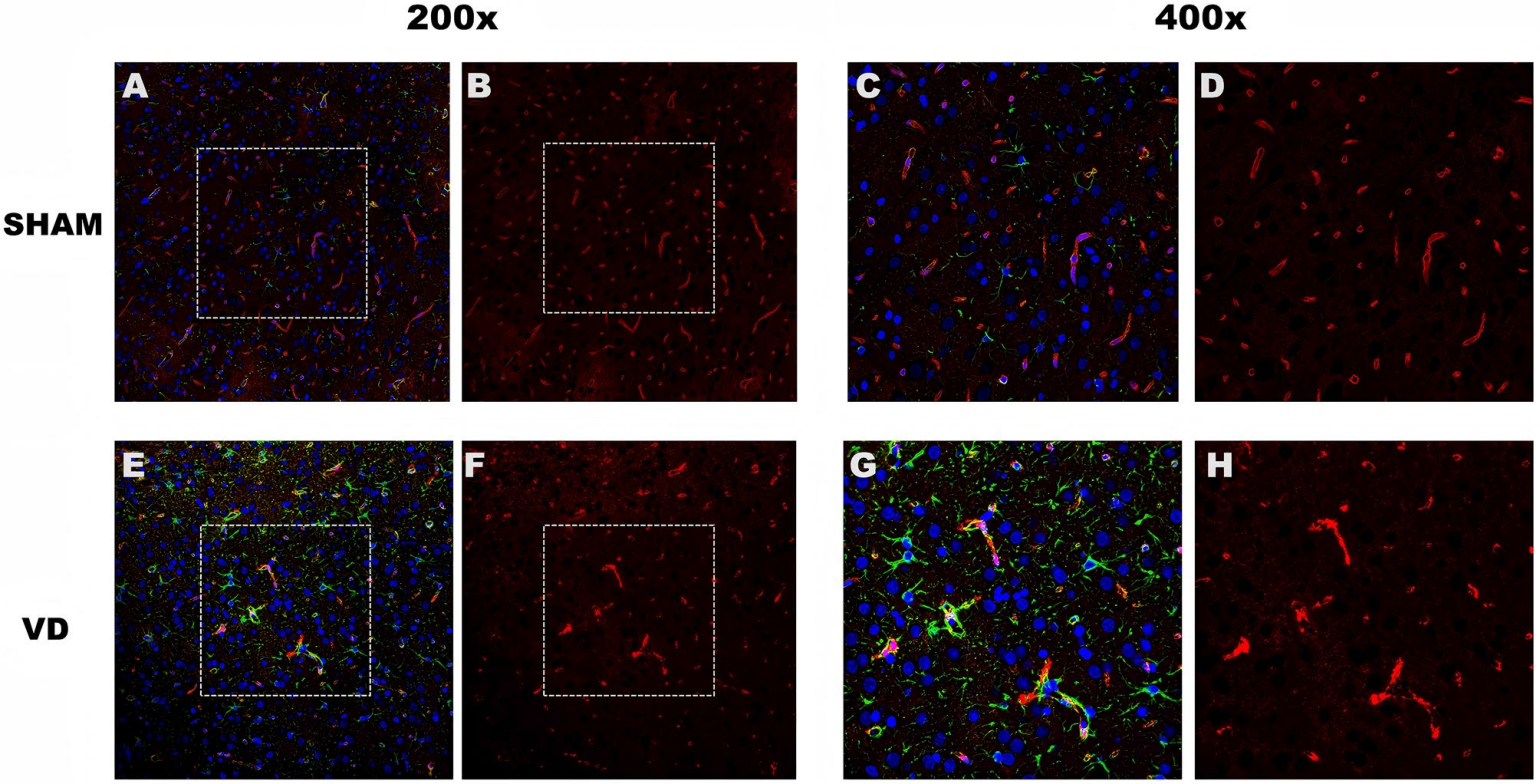
Immunofluorescence staining for AQP4 (red) and GFAP (green). Immunofluorescence staining of AQP4 with GFAP in cortical regions of the brain of (**A**) SHAM and (**E**) VD rats, under confocal microscopy at 200× magnification. (**B, F**) Representative plots of the fluorescence intensity of a single channel of AQP4 for figures **A** and **E**, respectively. (figures **C, D, G**, and **H**) Magnification of the dashed boxes in images shown in (**A**), (**B**), (**E**), and (**F**) at 400×

The ratio of the number of apoptotic neuronal cells in the hippocampal CA1 region to the change in signal intensity (%) on MRI at 5 h after contrast agent injection was analyzed using Pearson’s correlation analysis. This showed a strong linear correlation between the number of apoptotic neuronal cells in the hippocampal CA1 region (*r* = 0.8742, *p* < 0.001). The results of the HE staining experiments in the hippocampal CA1 region showing apoptotic neuronal cells and the structure of the hippocampus indicated that the damage characteristics were consistent with the MRI-determined characteristics of the impaired glymphatic system.

### Nissl staining

To examine hippocampal neuronal changes further, we stained rat brain sections at the hippocampal level with Nissl solution (Fig. 6). Neuronal cells in the CA1 region of the hippocampus in the VD group exhibited a loose and disorganized arrangement, with an expanded interstitial space between cells, a condensed nucleus, and intense cytoplasmic staining compared to the SHAM group (Fig. 6A–D). The number of Nissl bodies per microscope field were decreased in the VD group, whereas the SHAM group showed well-organized neurons in the CA1 area with uniform morphology, consistent neuronal gaps, visible Nissl bodies, and fewer apoptotic neuronal cells.

### Luxol fast blue staining

Brain sections at the level of the rat brain corpus callosum were stained with LFB to assess myelin content and distribution. The corpus callosum in the VD group (Fig. 6D) exhibited lighter staining and more disordered myelin, including myelin voids, than that in the SHAM group (Fig. 6C). In contrast, the SHAM group displayed well-aligned myelin, with no significant demyelination.

### Immunofluorescence staining

GFAP is a specific marker of glial cell activity and astrocytes. In the stained samples, the intensity and distribution of fluorescent signals reflect the expression of glial fibrillary acidic protein (GFAP). Figure 7 shows the results of the immunofluorescence staining for AQP4 and GFAP. The amount of AQP4 was decreased in the VD group (Figs. 7F and H), in contrast to that in the SHAM group (Fig.s 7B and D). Additionally, depolarization of AQP4 in the cerebral cortex of VD rats was higher than that in SHAM rats, and was accompanied by astrocyte proliferation.

## Discussion

In this study, we evaluated the impact of the glymphatic system function on VCI in a rat model. Diverse analytical approaches are employed in DCE-MRI studies of the glymphatic system. While some studies utilize pharmacokinetic modeling to estimate influx and clearance rate constants, providing deep mechanistic insights (Ringstad et al., JCI Insight 2018), the quantitative comparison of key functional parameters—such as time-to-peak (TTP) and the tracer retention index at a terminal time point—is an established and robust methodology for assessing glymphatic influx and clearance efficiency, respectively [37, 40]. Our study adopted this latter strategy, providing clear, quantitative evidence of dysfunction. We found that clearance rate of the CSF contrast agent, Gd-DOTA, from the brain interstitial space was slower in the VD than in the SHAM group, which was confirmed by fluorescence imaging of mouse brain sections. Behavioral tests showed a robust negative correlation between VCI and retention of Gd-DOTA contrast agents in the hippocampal region of VD rats. Critically, our quantitative DCE-MRI parameters could differentiate the VD from the SHAM group as early as 7 days post-modeling (the pre-clinical stage). The VD group exhibited a significantly delayed time-to-peak (TTP) (Hippocampus: VD 180 min vs. SHAM 120 min; *p* = 0.035) and a significantly elevated tracer retention index at 5 hours (Hippocampus: VD 24.82% vs. SHAM 12.86%; *p* < 0.05). These data indicate that glymphatic impairment appears earlier than measurable cognitive decline, suggesting its potential role as an early indicator for VCI. Pathology revealed a significant positive correlation between the number of apoptotic neurons in the hippocampal CA1 region and Gd-DOTA retention in the hippocampal region of VD rats. Thus, we show that ISF clearance via the glymphatic system was hindered in VD model rats, indicating that glymphatic system damage contributed to cognitive impairment in this group.

After Gd-DOTA injection via the medullary pool and using dynamic contrast-enhanced MRI for glymphatic imaging, we observed a gradual increase followed by a gradual decrease in signal intensity in the MR images of both VD and SHAM rats over time. The gradual increase in signal intensity after the injection of the contrast agent represents the influx of CSF into the brain parenchyma via the glymphatic system, mediated by AQP4 at the endfeet of astrocytes from the perivascular space, driven by the surface arterioles of the brain and perforating small arteries. As the concentration of the gadolinium contrast agent in the brain gradually increased, the signal intensity in the brain gradually increased. The gradual decrease after the signal had peaked represents the outflow process from the glymphatic system, that is, the process of CSF exchange with ISF and then flowing out of the brain into the perivenous interstitial space. As the gadolinium contrast agent in the brain was gradually removed, the signal intensity gradually decreased.

The blood-brain barrier (BBB) is composed of vascular endothelial cells interconnected by tight junction proteins, and is supported by astrocytes, pericytes, and the extracellular matrix [46]. Studies have shown that AQP4 interacts with α-syntrophin on astrocytic end-feet. This interaction not only regulates water transport but also indirectly contributes to the structural stability and functional maintenance of the BBB [47]. Consequently, AQP4 depolarization and glymphatic dysfunction may reflect a more fundamental compromise of neurovascular unit (NVU) integrity, potentially involving pericyte-mediated capillary dysfunction or aberrant endothelial cell signaling.

During the influx process, the VD group showed slower signal changes and later time to peak signal intensity than did the SHAM group, indicating impaired influx function of the glymphatic system in the VD group as compared to the SHAM group. Notably, this impairment in influx was also reflected in the significantly lower signal intensity observed in the VD group during the early phase (30–90 min post-injection), which likely indicates a slowed initial paravascular influx of the contrast agent. This early delay further supports the conclusion of compromised glymphatic inflow dynamics in VD rats. After peaking, the signal intensity of the VD group slowly decreased from approximately 3 h to the 5 h, while that of the SHAM group started to decrease after approximately 90 min to 2 h. Thus, compared to the SHAM group, the glymphatic system of rats in the VD group demonstrated slower clearance of Gd-DOTA and a higher residual amount of Gd-DOTA, resulting in a higher signal intensity, indicating that the efflux function of the glymphatic system was also impaired in the VD group as compared with that in the SHAM group.

The glymphatic system plays an important role in maintaining homeostasis in the intracerebral environment. In the VD model rats, the inflow and outflow functions of the glymphatic system were impaired to varying degrees. This impairment may be associated with weakened vascular pulsatility, a key driver of paravascular fluid motion. A reduction in this vascular drive could lead to obstruction and stagnation of CSF flow, which would be expected to impair the exchange of macromolecules and metabolites with the ISF. Consequently, the clearance of accumulated toxic substances via the glymphatic outflow may be compromised, potentially contributing to the development of cognitive impairment. Furthermore, chronic conditions such as low blood perfusion, cerebral ischemia-hypoxia, glutamate release, and oxidative stress may represent additional mechanisms that damage the nervous system and exacerbate cognitive impairment. We found no statistically significant difference in the target quadrant distance ratio or the target quadrant time ratio between the SHAM group and the VD group at 7 days (pre-VD), indicating a lack of difference in cognitive functioning between the VD and SHAM rats at 7-days postoperatively, and that neither group showed any significant impairment in spatial memory capacity. In contrast, the spatial memory capacity of the VD group was significantly worse than that of the SHAM group by 2 months after modeling, when the target quadrant distance and target quadrant time ratios were significantly lower in the VD group than in the SHAM group.

The results of the water maze experiments, combined with the results of the imaging experiments, demonstrated that the VD rat model at 7 days after modeling (corresponding to pre-VD clinically) did not show significant cognitive dysfunction, which only appeared by 2 months after modeling, when spatial memory function was impaired. However, 7 days after modeling, the VD model rats already showed differences in the MRI of the glymphatic system, and the impaired glymphatic system inflow and outflow functions of the VD rats model at 7-days post-modeling showed a strong linear correlation with water maze results at 2-months post-modeling. Therefore, early glymphatic system function can act as a predictor of VD development.

The HE staining results showed that the VD group had loosely arranged neurons, a higher number of apoptotic cells, and increased cellular gaps in the hippocampal region than did the SHAM group, indicating an impaired hippocampal structure in VD rats. After surgery, the cerebral vascular pulsatility of the rats was impaired, CBF was decreased, the flow in the perivascular interstitial space was obstructed, more CSF was retained in the perivascular interstitial space, and clearance of metabolites from the brain was impaired.

AQP4, which is enriched on the endfeet of perivascular astrocytes, is an important component of the glymphatic system, facilitating the passage of CSF from the peri-arterial space into the interstitium and assisting in the removal of solutes from the interstitium by convection [48]. Nearly 70% of mice with reduced glymphatic system function have an AQP4 gene knockout. Impairment of the glymphatic system may occur with a decrease in AQP4 levels. The concept of AQP4 depolarization has been proposed, whereby a reduction in AQP4 levels leads to a decrease in its colocalization with perivascular astrocyte peduncles. The absence of co-localization of AQP4 with blood vessels severely affects the function of the glymphatic system [49].

Dual-labeling immunofluorescence of AQP4 and GFAP showed an increase in AQP4 depolarization after 7 days of modeling (corresponding to the preclinical stage of vascular dementia), which led us to conclude that depolarization of AQP4 by 7 days after modeling (pre-VD) is likely to be responsible for the impaired functioning of the glymphatic system in the brain.

The quantitative analysis presented here, based on TTP and the terminal retention index, robustly supports our conclusions. However, we recognize that pharmacokinetic modeling of the entire time-series data could provide additional insights into precise influx and clearance rates. Future studies incorporating such modeling would be a valuable extension of our work, building upon the clear predictive relationship we have established here.

Further studies are needed to investigate the specific mechanism of AQP4 depolarization, which may provide new perspectives for the prevention and treatment of VCI.

## Conclusion

In the VD group, the clearance rate of the CSF contrast agent Gd-DOTA in the interstitium of the hippocampus, cortex, and olfactory bulb was slower than that in the SHAM group, which was also corroborated by the fluorescence imaging analysis. Strong negative correlations were found between residual amounts of the Gd-DOTA contrast agent and a fluorescent tracer in the hippocampus of the VD group, and cognitive impairment detected in behavioral tests, which suggested that VD hinders interstitial fluid clearance in the hippocampus and that this impairment of the glymphatic system significantly contributes to the cognitive deficits caused by VD.

Additionally, our study showed that whole-brain MRI offers a sensitive and non-invasive method for quantitatively evaluating the exchange of CSF and ISF in VD, which shows promise for clinical application in this and potentially other neurological conditions.

## Electronic supplementary material

Below is the link to the electronic supplementary material.

Supplementary material 1

## Data Availability

We express our sincere gratitude for the editor's diligent attention and acknowledgment of our research endeavors. We fully acknowledge the significance of data sharing as stipulated by SCI journals. However, due to the policies and confidentiality agreements within our laboratory, we are unfortunately unable to provide the raw data. Nevertheless, we have meticulously described the experimental design, analysis, results, and the procedures employed for data analysis and processing. Should the editor and reviewers have further questions regarding specific data, we are committed to providing detailed explanations and clarifications.
