# Supplementary figures and images for: Impact of early glymphatic disorders on the development of vascular dementia
Source: BMC Med Imaging. 2026 Mar 16;26:209. doi: 10.1186/s12880-026-02284-5 (PMC13104415; doi:10.1186/s12880-026-02284-5)

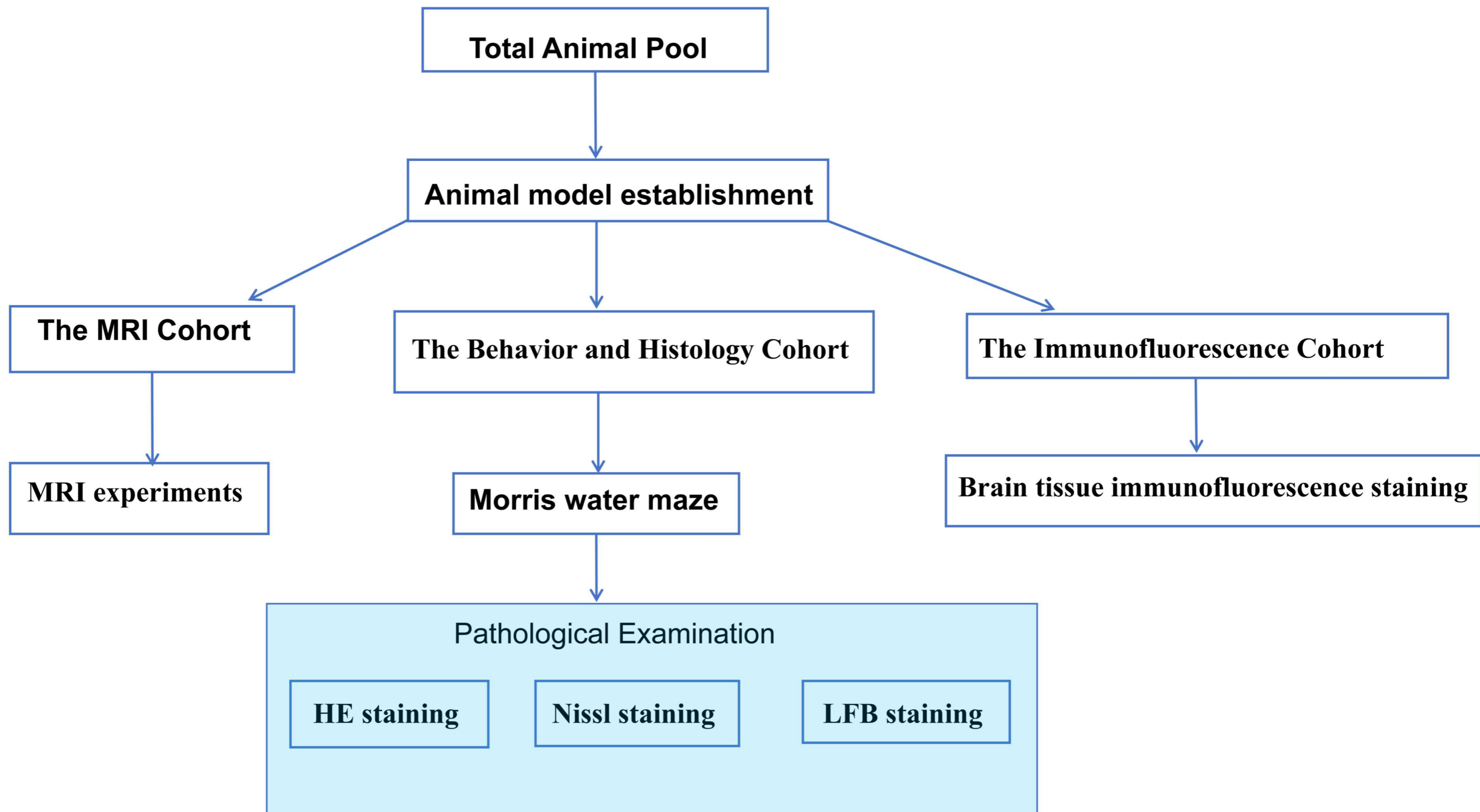

Supplement: Supplementary file 1 — Supplementary material 1 [file 12880_2026_2284_MOESM1_ESM.zip › 12880_2026_2284_MOESM2_ESM/Supplementary Figure 1.pdf]

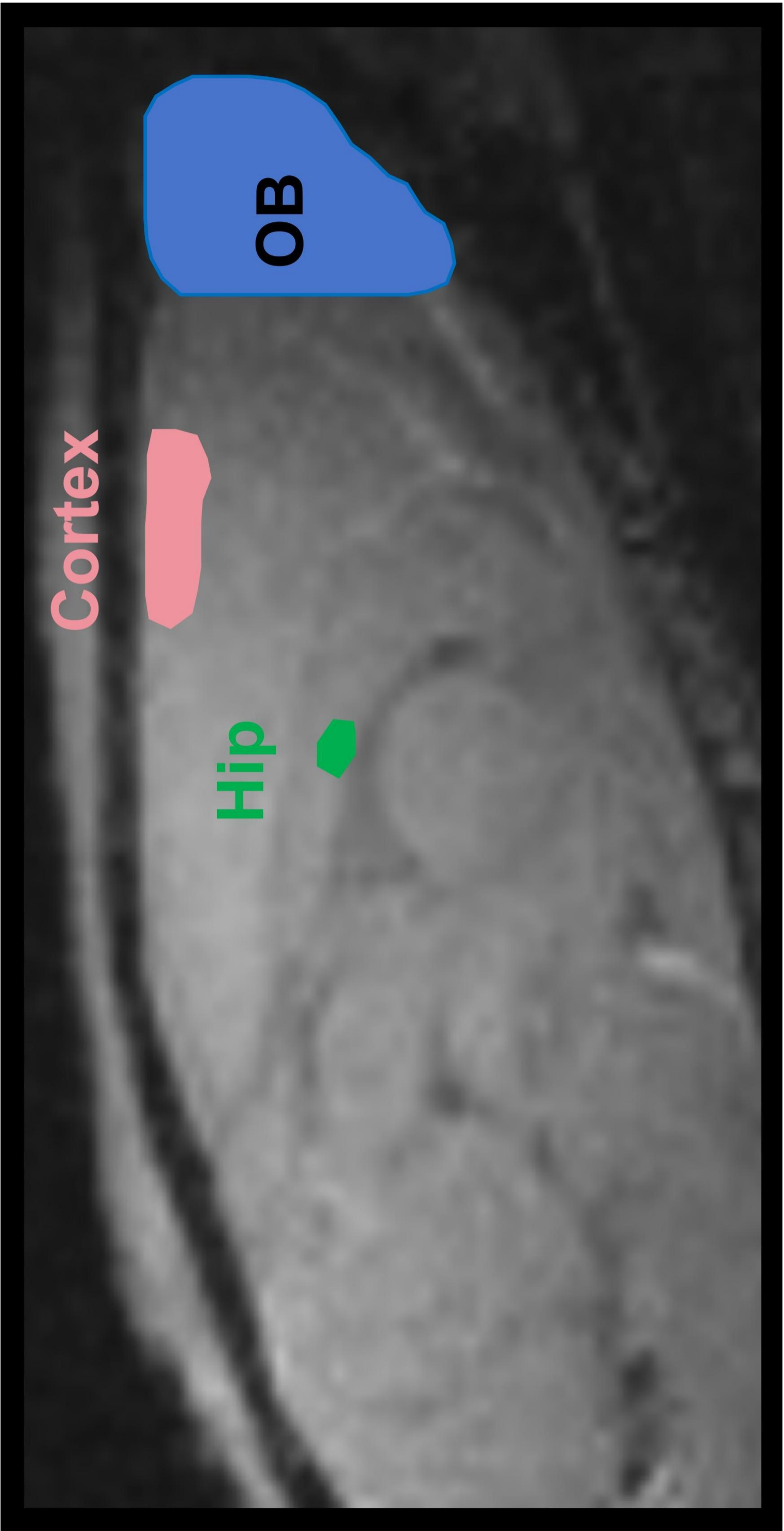

Supplement: Supplementary file 1 — Supplementary material 1 [file 12880_2026_2284_MOESM1_ESM.zip › 12880_2026_2284_MOESM2_ESM/Supplementary Figure 2.pdf]
